# The environment and dry eye—manifestations, mechanisms, and more

**DOI:** 10.3389/ftox.2023.1173683

**Published:** 2023-08-23

**Authors:** Sneh Patel, Rhiya Mittal, Naresh Kumar, Anat Galor

**Affiliations:** ^1^ Division of Physical Medicine and Rehabilitation, Veterans Affairs (VA) Greater Los Angeles Healthcare System, Los Angeles, CA, United States; ^2^ University of California Los Angeles (UCLA), Los Angeles, CA, United States; ^3^ University of Miami Leonard M. Miller School of Medicine, Miami, FL, United States; ^4^ Bascom Palmer Eye Institute, University of Miami, Miami, FL, United States; ^5^ Department of Public Health Sciences, University of Miami, Miami, FL, United States; ^6^ Ophthalmology and Research Services, Miami VA Medical Center, Miami, FL, United States

**Keywords:** dry eye disease, ocular surface pain, environment, toxicology, temperature, humidity, air pollutant, allergy

## Abstract

Dry eye disease (DED) is a multifactorial condition that often presents with chronic symptoms of pain (that can be characterized as “dryness,” “burning,” and “irritation,” to name a few) and/or fluctuating or poor-quality vision. Given its multifactorial nature, several pathophysiologic mechanisms have been identified that can underlie symptoms, including tear film, ocular surface, and/or corneal somatosensory nerve abnormalities. Research has focused on understanding how environmental exposures can increase the risk for DED flares and negatively impact the tear film, the ocular surface, and/or nerve health. Given that DED is a common condition that negatively impacts physical and mental functioning, managing DED requires multiple strategies. These can include both medical approaches and modulating adverse environmental conditions, the latter of which may be a cost-effective way to avoid DED flares. Thus, an understanding of how environmental exposures relate to disease is important. This Review summarizes research on the relationships between environmental exposures and DED, in the hope that this information will engage healthcare professionals and patients to consider environmental manipulations in their management of DED.

## 1 Introduction

Dry eye disease (DED) is a common ocular condition and a major cause of chronic ocular surface pain and/or fluctuating and poor-quality vision. It is a multifactorial condition, characterized by tear film instability, high or unstable osmolarity, ocular surface inflammation, and/or somatosensory abnormalities. DED does not have a “gold standard” definition, but instead is often referred to as an umbrella term under which various disease phenotypes fit (Galor et al., 2020; Villani et al., 2020). Given this complexity, it is not surprising that heterogeneity exists with respect to the pathophysiological pathways underlying the disease (Craig et al., 2017; Ganesalingam et al., 2019). Of these, this Review will focus on how environmental exposures may impact DED onset, severity, and persistence.

This Review is needed as less is known about the relationships between DED and adverse environmental exposures compared to other disease contributors. For example, T-cell-mediated inflammation has been studied in individuals with DED and comorbid Sjögren’s syndrome (SS), neurovascular instability has been examined in individuals with DED and rosacea, and neuropathic mechanisms have been probed in individuals with DED and comorbid migraine or fibromyalgia. Other studies have focused on behavioral factors (e.g., contact lens use and smoke exposure) and medications (e.g., antihistamines, antidepressants, and antihypertensives) as they relate to DED.

In comparison to these established relationships, less is known about the etiology of DED in response to adverse environmental conditions. Given that DED impacts physical and mental functioning, understanding the factors that contribute to the disorder is essential and can help providers improve care algorithms and deliver precision medicine. Furthermore, certain environmental manipulations may be more cost-effective than medical therapy in controlling severe and/or refractory symptoms. This Review will summarize the current knowledge on the toxicological mechanisms of environmental exposures as they relate to DED manifestations.

## 2 Body

### 2.1 Symptoms and signs of DED

When examining studies on DED, it is important to understand the constellation of symptoms and signs that fall under the disorder. The diagnosis of DED is made by clinical examination, based on the presence of symptoms (e.g., that can be assessed with various validated questionnaires), slit lamp findings, and in-clinic point-of-care tests. Given that different risk factors may relate to different aspects of DED, it is important to examine disease definitions when reviewing epidemiological studies on DED.

For symptoms, ocular surface pain is a common complaint patients present with, with common descriptors that include “dryness,” “burning,” “aching,” and “tenderness,” to name a few. Pain symptoms can arise from nociceptive sources (activation of nociceptors due to abnormalities in peripheral tissues), neuropathic sources (abnormalities in somatosensory pathways to and from the cornea), or a combination of both (Stucky et al., 2001; Basbaum et al., 2009). Ocular surface pain, whether secondary to DED or other causes, is a major source of morbidity, and DED-associated chronic ocular surface pain is a leading cause of ophthalmic healthcare costs (Yu et al., 2011) and has deleterious effects on the quality of life and productivity (Goyal and Hamrah, 2016; Patel et al., 2019). Considering all symptoms of DED (pain and visual symptoms), cost-of-illness analyses have estimated the burden of DED at nearly $3.84 billion, including indirect costs (loss of work) (Yu et al., 2011).

Ocular surface pain can be quantified using standardized questionnaires, each aimed at eliciting different characteristics of pain. For example, the 5-Item Dry Eye Questionnaire (DEQ-5) assesses the frequency and intensity of dryness and discomfort, along with the frequency of tearing (Chalmers et al., 2010). The Ocular Surface Disease Index (OSDI) assesses the frequency of sensitivity to light, grittiness, and painful or sore eyes, along with visual symptoms, triggers, and quality of life implications (Schiffman et al., 2000). Pain-specific questionnaires have also been developed, most of which use a Likert-type Numerical Rating Scale (NRS), including the Ocular Pain Assessment Survey (OPAS; intensity, descriptors, and quality of life) (Qazi et al., 2016) and the Neuropathic Pain Symptom Inventory modified for the Eye (NPSI-E), the latter of which focuses on neuropathic descriptors of eye pain (Farhangi et al., 2019).

“Signs” of the disease are examined with in-clinic tests that assess ocular structure and function, with certain thresholds used as cut-offs for the clinical diagnosis of DED. These include tests that look for alterations in tear stability (e.g., tear breakup time (TBUT)) and production (e.g., Schirmer’s test, with or without anesthesia) and structural integrity (e.g., corneal and conjunctival staining using vital dyes such as fluorescein, lissamine green, and Rose Bengal), and assess corneal function and structure (Mehra et al., 2020; Patel and Sarantopoulos, 2023). Corneal function can be evaluated via corneal sensitivity (qualitatively assessed in the clinic with a cotton tip or floss or quantified in the research arena with an esthesiometer). Structural attributes are examined microscopically via *in vivo* confocal microscopy (IVCM); commonly reported findings include the presence of immune cells within the cornea (e.g., dendritic cells, most commonly noted in individuals with aqueous tear deficiency (ATD) in the setting of auto-immune disease) and corneal nerve abnormalities (e.g., decreased nerve density and increased nerve tortuosity, also common in individuals with systemic auto-immune diseases such as SS) (Hwang et al., 2021).

### 2.2 Environmental health risks

Risk relationships with environmental factors have been studied for several ocular and systemic conditions (Paschides et al., 1998; Syndulko et al., 1996; D’Amato et al., 2015; Michelozzi et al., 2009). Generally, studies classify exposures as “indoor” or “outdoor” (also known as ambient) when reporting associations. Studies on outdoor exposure are more common, even though we spend most of our time indoors, at least partially due to the availability of national ambient meteorological databases. Some commonly studied factors include air pollutants (e.g., ozone, O_3_; nitrogen dioxide, and NO_2_), aeroallergens (e.g., pollen, dander, mold, and dust), meteorological conditions [e.g., temperature and relative humidity (RH)], interaction effects (e.g., the effect of temperature and RH simultaneously, also known as heat stress), and behavioral factors (e.g., exposure to smoke, chemicals, medications, and contact lens use). It is important to note that studying the environment is challenging, regardless of the type of exposure—the study of ambient variables requires the integration of patient health data and environmental data with different spatiotemporal scales, which can result in exposure uncertainty (Kumar, 2016). On the other hand, accurate measurements of indoor variables can require special devices like climate control chambers (Calonge et al., 2018) to control indoor exposures or a special set-up to measure exposures.

### 2.3 Outdoor environment

#### 2.3.1 Temperature

Perhaps the least studied of all ambient variables, toxic exposure to temperature is thought to mainly affect ocular health by its influence on the tear film (Nagymihályi et al., 2004). Controlled chamber studies have described the direct impact of temperature on the tear film. Specifically, two controlled environment chambers in Europe (Abusharha and Pearce, 2013; Abusharha et al., 2016) exposed individuals to increasing ambient temperatures at constant RH and found that tear film parameters varied by temperature level. One study found that lipid layer thickness increased with increasing temperature (20–40 nm at 5°C and 10°C vs. 40–90 nm at 15°C, 20°C, and 25°C; *p* < 0.05) (Abusharha and Pearce, 2013). Similar findings were noted in the second study (median lipid layer thickness 20–40 nm at 5°C and 10°C vs. 40–90 nm at 15, 20, and 25°C; *p* < 0.005), but interestingly, this second study also found that the evaporation rate increased with temperature (0.06 μL/min at 5°C vs. 0.17 μL/min at 25°C; *p* < 0.005) (Abusharha et al., 2016). These findings are difficult to interpret, as other studies found that a thicker lipid layer led to a lower evaporation rate, thus having a protective effect on the ocular surface (Craig and Tomlinson, 1997; Giraldez et al., 2009). As such, further research is needed to understand how temperature impacts the risk of ocular surface disorders like DED, beyond its effects on lipid thickness.

On an epidemiological level, associations between temperature and DED have varied. A Taiwanese study of 25,818 subjects with known DED (not further defined) found that temperature was positively associated with a DED diagnosis (β = 1.01, 95% CI = 1.00 to 1.02; *p* < 0.001). In this model, RH had an inverse relationship with DED (β= 0.93, 95% CI = 0.91 to 0.95; *p* < 0.001), and NO_2_ had a positive relationship (β = 1.08, 95% CI = 1.04 to 1.11; *p* < 0.001) (Zhong et al., 2018). In comparison, a Taiwanese study of 351 patients with known DED (OSDI ≥ 13, TBUT ≤ 5, staining) reported that temperature was inversely related to symptoms (via OSDI; β = −0.84, 95% CI = −1.34 to −0.33; *p* < 0.005) and tear production (Schirmer’s: β= −0.73, 95% CI = −1.19 to −0.26; *p* < 0.005) (Ho et al., 2022). Further highlighting the variable findings on temperature, an American study that examined 3.41 million visits at Veteran Affairs (VA) eye clinics between July 2006 and July 2011 found that DED (via ICD9 code; diagnosed in 17.4% of the study population) was most frequently diagnosed in the winter and spring months, compared to the fall and summer (18.7% ± 0.98% and 18.5% ± 4.16%, respectively), with the highest frequency occurring in April (20.9% ± 0.14%) (Kumar et al., 2015). These data suggest that factors beyond temperature alone may impact DED presentation.

In summary, while there is evidence suggesting that the tear film and lipid layer are affected by temperature extremes, the findings are inconsistent as to which extreme of the temperature scale is most harmful. These findings may suggest that the relationship between temperature and DED is non-linear and is instead possibly a “U”-shaped curve, with a “Goldilocks” zone (temperatures below or above this zone having a detrimental impact on tear film health). In fact, the American Society of Heating, Refrigerating, and Air-Conditioning Engineers has recognized the concept and recommended that the indoor temperature be set between 20°C and 25°C (Abdul-Wahab et al., 2015). Further studies are necessary to understand and translate this recommendation to individuals with DED.

Exposure to temperature change is another important factor that may relate to the risk of DED. Studies examining temperature change often utilize the diurnal temperature range (DTR) as a measure of change, which measures the difference between the maximum and minimum daily temperature. A higher DTR has been reported as a risk factor for disease flares across several conditions, from asthma (Xu et al., 2013; Kim et al., 2014; Qiu et al., 2015; Li et al., 2016) to heart failure (Lim et al., 2012). It is hypothesized that exposure to abrupt temperature change may impact the function of immune cells involved in inflammatory and allergic presentations, specifically through altered release of cytokines and cytotoxic proteins (Lobefalo et al., 1999; Graudenz et al., 2006). The aforementioned American study, which studied visits to 3.41 million VA eye clinics across the United States between July 2006 and July 2011, reported on this association with respect to DED—the study found that change in temperature had more influence on DED presentation than absolute temperature throughout each season. The greatest decrease in symptom intensity (via OSDI, DEQ5, and NPSI-E) occurred in winter and summer, when the weather change from the previous season was less abrupt, compared to spring and autumn. The study hypothesized that abrupt meteorological changes may have a detrimental effect on the lacrimal unit (Kumar et al., 2015); however, further studies are necessary to test this hypothesis.

#### 2.3.2 Relative humidity (RH)

Low RH (e.g., desiccating stress) is a well-described risk factor for DED (Smith, 2007). Adequate production and stability of tears is essential to a healthy tear film, and any destabilization in these variables can lead to ocular surface diseases like DED. While the pathway is not entirely understood, studies have implicated a negative association between RH and tear osmolarity (e.g., induction of stress via a hyperosmolar mechanism) (González-García et al., 2007) and alterations in protein oxidation within the tear film and Meibomian lipids (Abusharha and Pearce, 2013) as potential causes of this relationship. In addition to this, RH has also been shown to directly affect tear film evaporation; specifically, aridity affects vapor concentration, which, together with tear film thickness, determines the evaporative flux at the ocular surface (Peng et al., 2014). In this manner, RH may also exert its effects on tear film health by influencing the tear evaporation rate, a finding described in several studies.

Describing these effects, one of the previously described European climate chamber studies also reported on the relationship between RH and tear dynamics. This study examined two conditions: RH set at 40% (normal) and at 5% (desiccating stress). Tear film abnormalities noted in the desiccating (low RH) environment included an increase in tear evaporation, a decrease in tear production, a decrease in lipid layer thickness, and an increase in ocular pain (specific data not provided; *p* < 0.05 for each) (Abusharha and Pearce, 2013). Supporting these human findings, a mouse study reported that exposure to low RH (RH = 18.5% ± 5.1%) for 28 days after an initial equal exposure to normal RH (RH = 50%–80%) led to decreased tear production via cotton thread wetting (baseline: ∼2.2 ± 0.2 mm; day 3: 1.4 ± 0.3 mm; *p* < 0.005; day 28: 1.3 ± 0.4 mm; *p* < 0.05) and increased fluorescein staining (baseline: ∼1.5 ± 1.5; day 3: 5.8 ± 2.2; *p* < 0.0001; day 28: 4.6 ± 2.3; *p* < 0.05) (Barabino et al., 2005). However, not all studies reported an inverse relationship between DED and RH—one English study of 10 individuals with mild–moderate DED (symptoms, TBUT<10 s, Schirmer<10 mm) and 10 controls exposed groups to varying RH (5%, 40%, and 70%, for 25 min on 3 separate days). As the RH increased from 5% to 70%, tear evaporation rates linearly decreased in both groups (∼100 g/m^2^/hr at 5%, ∼70 g/m^2^/hr at 40%, and ∼0 g/m^2^/hr at 70% for DED vs. ∼90 g/m^2^/hr at 5%, ∼40 g/m^2^/hr at 40%, and ∼0 g/m^2^/hr for controls; *p* < 0.05 between points in each group, respectively), supporting the results of the previous studies. However, tear stability (TBUT) followed a U-shaped curve in both groups with varying RH (4.90 ± 1.66 s at 5%, 6.31 ± 2.21 s at 40%, and 5.90 ± 1.91 s at 70% in the DED group vs. mean 17.80 ± 3.91 s at 5%, 20.70 ± 5.88 s at 40%, and 20.00 ± 5.35 s at 70% in controls), suggesting an optimal value at 40% RH (Madden et al., 2013).

Many epidemiological studies have noted relationships between DED and RH. A Taiwanese study of 25,818 subjects diagnosed with DED found that lower RH (β = 0.93, 95% CI = 0.91 to 0.95; *p* < 0.001) was associated with DED diagnosis, along with temperature and NO_2_ (Zhong et al., 2018). A Korean study of 16,824 participants from January 2010 to December 2012 found an inverse relationship between RH and DED symptoms (OR = 0.87; 95% CI = 0.77 to 0.98; *p* = 0.03) and diagnosis (OR = 0.86; 95% CI = 0.76 to 0.97; *p* = 0.01) (Hwang et al., 2016). Supporting this association, a Chinese case-crossover study of 5,062 individuals diagnosed with DED found that lower RH was associated with an increased risk for an outpatient DED diagnosis visit (specific data not provided; *p* < 0.05) (Mo et al., 2019). However, just as observed with the chamber studies, not all epidemiological studies have reported an inverse relationship—one American study of 97 individuals who underwent indoor RH monitoring instead found that RH was positively associated with symptoms (OSDI: r = 0.30, 95% CI = 0.07 to 0.49; *p* = 0.01) and Meibomian gland (MG) dropout (r = 0.27, 95% CI = 0.05 to 0.47; *p* = 0.02), and negatively associated with tear production (Schirmer: r = −0.25, 95% CI = −0.45 to 0.02; *p* = 0.03) (Huang et al., 2020). Of note, the group hypothesized that the noted association between RH and DED was not driven by RH alone, but by the interaction between RH and particle size via the hygroscopic effect (the ability of particulate matter (PM) to absorb water and increase in size under high RH). These findings suggest that, like with temperature, a U-shaped curve may describe the relationship with RH. In fact, the Environmental Protection Agency recommends an ideal RH level of 30%–50% (Wendt et al., 2004), providing credence to a potential “Goldilocks” zone.

#### 2.3.3 Air pollution

Air pollutants can be divided into airborne PM and gas molecules, both of which are generated by indoor and outdoor sources, such as fossil combustion (e.g., automobile emissions and aerosolization of cooking and cleaning products) (Mandell et al., 2020). Air pollutants of special interest to ocular health, as outlined by the World Health Organization (WHO), are O_3_, NO_2_, sulfur dioxide (SO_2_), carbon monoxide (CO), and PM (Versura et al., 1999; Jung et al., 2018).

Air pollutants are hypothesized to impact ocular and periocular components variably, depending on their composition. While all types act as sources of inflammation, ultrafine PM particles can cross the corneal epithelium to induce stress in deeper layers of the eye, while larger particles can settle upon and physically damage (e.g., abrasion) the ocular surface and periocular lid margin (Mandell et al., 2020). Gaseous pollutants, including reactive gases [e.g., NO_2_, SO_2_, O_3_, and volatile organic compounds (VOCs)], react with the tear film and induce a local stress reaction (Mandell et al., 2020). Several mechanisms have been postulated, including the formation of direct irritant reagents at the ocular surface (e.g., solubilization of sulfur-containing compounds to create sulfurous or sulfuric acids) and activation of conjunctival antigen-presenting cells, leading to a pro-inflammatory response (Jung et al., 2018).

One climate control study focused on air pollutants and symptoms and signs of DED in humans and found a decrease in tear stability (via TBUT) after exposure. Specifically, a Danish study exposed 10 individuals to clean (41 μg/m^3^ dust) and polluted air (394 μg/m^3^ dust) in a randomized order for 3 h and reported a decrease in TBUT compared to baseline (specific data not provided; *p* < 0.05) (Pan et al., 2000). Epidemiological studies have consistently reported positive relationships between air pollution and DED. The Chinese case-crossover study of 5,062 individuals with DED identified that same-day exposure to PM_2.5_ (OR = 1.02, 95% CI = 1.01 to 1.03; *p* < 0.01) and PM_10_ (OR = 1.01, 95% CI = 1.003 to 1.02; *p* < 0.01) was a risk factor for a DED diagnosis visit, along with decreasing RH (Mo et al., 2019). Similarly, the Taiwanese study of 25,818 subjects with DED found that increasing NO_2_ (β = 1.08, 95% CI = 1.04 to 1.11; *p* < 0.001) was associated with a DED diagnosis, along with ambient temperature and RH (Zhong et al., 2018). In a similar fashion, a Korean study of 16,824 participants found positive relationships between O_3_ levels with DED symptoms (OR = 1.16; 95% CI = 1.02 to 1.30; *p* = 0.04) and DED diagnosis (OR = 1.21; 95% CI = 1.05 to 1.40; *p* = 0.008) (Hwang et al., 2016). Finally, a prospective Korean study of 43 patients with DED undergoing treatment (symptoms, TBUT, staining) noted that O_3_ (β = 0.33, 95% CI = 0.16 to 0.49; *p* < 0.001) and PM_2.5_ (β = 0.38, 95% CI = 0.06 to 0.70; *p* < 0.02) levels were positively associated with symptoms (via OSDI), while PM_10_ (β = −0.03, 95% CI = −0.045 to −0.01; *p* = 0.001) was negatively associated with tear stability (TBUT) (Kim et al., 2020).

In summary, studies have overwhelmingly reported a positive association between exposure to different outdoor air pollutants and various aspects of DED.

#### 2.3.4 Airborne allergens

While allergy and DED are separate entities, DED is often comorbid with “ocular allergy” (Leonardi et al., 2021), and DED flares can occur as a result of exposure to allergens, both seasonally and perennially (Friedlaender, 2011; Ortega et al., 2022). One systematic review reported that ∼50% of individuals with allergic conjunctivitis (AC) have comorbid DED, and ∼20% of individuals with DED have comorbid AC (Akasaki et al., 2022). Other studies have found molecular links between DED and AC—an American study on 75 patients with symptoms or signs of DED reported that 17% of subjects (13/75) had high tear IgE (>1 ng/mL) and that this group was more likely to be exposed to allergens in their home (e.g., pets: OR = 11.5; *p* = 0.002; smoke: OR = 38.6; *p* = 0.008), supporting the idea of an allergic component to DED in some individuals (Dermer et al., 2019). Shared signs have also been noted between DED and allergy, for example, corneal epithelial disruptions assessed with Rose Bengal and fluorescein (Dogru et al., 2008). Overall, these findings suggest that allergens may impact various aspects of ocular surface health, including tear stability, mediators of inflammation, and mucin abnormalities, leading to sign overlap with DED.

No chamber studies have examined the association between allergens and DED, but epidemiological studies have reported positive links between ocular allergy and DED. In a Swedish study of 89 children aged 7–18 with pollen allergy (positive skin prick test or presence of IgE), ocular pain scores (pain Likert 0–3) increased linearly with pollen grain exposure over 42 days, until exposure to 150 grains/cm^3^, where the trend flattened (specific data not provided; *p* < 0.05) (Kiotseridis et al., 2013). In addition to pollen, studies have also examined mold spores, which have been classified as aeroallergens and as bioaerosols across different studies. In a study of 3,485 adults in China, individuals who lived in homes with more signs of mold (severity score quantified by the presence of mold/damp stains, moldy odor, dampness on bed/clothing, window pane condensation in winter, and water damage) had an increased risk of ocular pain compared to those who lived in homes with fewer signs (OR = 3.20, 95% CI = 1.67 to 6.15; *p* < 0.01) (Lu et al., 2016).

Overall, studies suggest a positive relationship between allergens and DED, most notably pain and tear stability. Of interest, environmental studies focusing on allergies have coincided with findings reported for DED—allergic diagnoses and symptoms have been positively linked to temperature, negatively to RH, and positively to air pollution (Reinikainen et al., 1992; Mendell et al., 2002; Wolkoff et al., 2003; Rozanova et al., 2009; Idarraga et al., 2020). Further studies are needed to examine the overlapping pathophysiology between allergy and ocular surface disease and their relationships to the environment.

#### 2.3.5 Atmospheric pressure

Although not as well-studied, atmospheric pressure may also impact ocular health. Atmospheric pressure is especially important in high-altitude areas (e.g., mountainous regions and aircrafts mid-flight), where its value decreases (the amount of gas molecules in the air decreases, making the air less dense than that closer to the ground), as it is hypothesized that lower atmospheric pressure leads to increased tear film evaporation (Tesón et al., 2013). Supporting this idea, the previously discussed American VA-based study found that atmospheric pressure was a risk factor for a DED diagnosis—the risk of a DED diagnosis was 13% higher in patients residing in regions where atmospheric pressure was 1 standard deviation higher than the population mean (incidence rate ratio (IRR) = 1.13, 95% CI = 1.129 to 1.133; *p* < 0.001) (Galor et al., 2014). Further studies are needed to examine this association and to develop appropriate mitigation strategies.

#### 2.3.6 Bioaerosols

Bioaerosols are small biological particles (0.001–100 μm in diameter) that are present in both ambient outdoor and indoor air and are characterized as another form of air pollutant in some studies. These molecules originate from endotoxins, glucans, mycotoxins, allergens, bacteria, and fungal spores and are made airborne by the handling of industrial/agricultural products (soil, plants, animals, etc.). Similar to other airborne particles (PM and allergens), concentrations of bioaerosols vary by meteorological conditions, seasonality, and by human and animal activity (Rock et al., 2021).

It is hypothesized that lipolytic enzymes and polar lipids secreted by eyelid-colonized bacteria may influence meibum composition and health, and thus relate this exposure to the risk of surface disorders like DED (Dougherty and McCulley, 1986). Unfortunately, there is a large paucity of studies that focused on this relationship, with only a few epidemiological studies having been conducted. Only one study has specifically examined bioaerosols in the context of DED—another Australian study obtained swabs from the inferior conjunctival fornix and lid margin of 66 individuals with DED (symptoms, TBUT <10s, staining >3 on Oxford) and 18 controls and found more colony-forming units (CFUs) in the DED vs. the control group (106 ± 82 CFUs vs. 12 ± 18 CFUs; *p* < 0.0001). Moreover, within the DED group, individuals with (*n* = 15) versus without (*n* = 51) MG dysfunction (eyelid thickening, irregularity, telangiectasia, gland loss, capping, or abnormal meibum) had higher CFUs on average (95 ± 66 CFUs vs. 12 ± 18 CFUs; *p* < 0.05) (Albietz and Lenton, 2006).

Given the lack of data, further studies examining both the relationship between DED and airborne bioaerosols as well as the molecular mechanism of injury are necessary. This is especially important given that there are no existing recommended indoor, outdoor, or occupational bioaerosol concentration standards in the United States.

#### 2.3.7 Important considerations for outdoor variables

While we have summarized studies examining the relationships between environmental factors and DED, there are considerations to keep in mind when analyzing these results. First, environmental factors affect one another, making it difficult to analyze the effect of an individual exposure with respect to ocular diseases like DED (D’Amato et al., 2015; Vocks et al., 2001; Pfab et al., 2010; Hong et al., 2016; Levetin and Van de Water, 2008; Ju et al., 1998; Mimura et al., 2014; Park et al., 2020). For example, higher temperatures can promote aeroallergen dispersion—some genes that encode pollen production work in a heat-dependent manner; thus, increasing temperature can promote the earlier initiation of flowering and enhanced allergenicity of aeroallergens (Ju et al., 1998; Levetin and Van de Water, 2008). Similarly, low RH can promote the suspension of airborne pollutants (PM_2.5_ and PM_10_) and airborne pollen levels (Wyon et al., 2002; Qiu et al., 2019). As previously discussed, RH can also impact PM size, known as the hygroscopic effect (PM can absorb airborne moisture in settings of high RH and inflate in size) (Huang et al., 2020). These confounding factors must be taken into consideration when examining the reported relationships between RH and ocular disease.

Second, population demographics vary across climate regions and may play a role in environmental susceptibility; this may impact comparisons across geographically diverse studies. For example, heat sensitivity is heightened in elderly women, patients with decreased mobility or dementia, those on medications that affect thermoregulation (diuretics or anticholinergics), and those with disorders that compromise thermoregulation (obesity, hypertension, pulmonary disease, and diabetes) (Kovats and Hajat, 2008; Kenny et al., 2010). In addition, individual differences in the ability to adapt to one’s environment may drive geographic differences (Hori et al., 1977). A Japanese study found that men in hot subtropical zones who later moved to colder temperate zones showed signs of superior heat acclimation than those who spent their lives in the temperate region, including less skinfold thickness (e.g., upper arm: 5.3 ± 2.3 mm vs. 7.7 ± 3.2 mm; *p* < 0.001) and more effective sweating with less salt wasting (0.022 ± 0.004 mEq/L vs. 0.029 ± 0.008 mEq/L; *p* < 0.05) (Hori et al., 1978). Several biologic modifications underlie climate adaptation, including a heat-dependent shearing mechanism for controlling blood flow (Carter et al., 2014), improved fluid balance and sweating mechanics (Périard et al., 2015), and changes in thermal behavior (e.g., brown adipose plasticity and metabolic enzyme activity) (Lee et al., 2014; Ning et al., 2016), and it is not known how these factors impact tear metrics, corneal epithelial cells, and corneal nerves. These factors may confound study results and account for variability across studies, along with other factors such as DED definitions and variance in methods for capturing environmental exposures.

### 2.4 Indoor environment

The indoor environment is also an important potential contributor to DED (Mandell et al., 2020). Ocular irritation is a frequently reported complaint of office workers, with studies suggesting that beyond indoor meteorological exposures, activities like work-related tasks (concentration causing decreased blink rate) and behavioral factors (contact lenses, eye make-up, medications, and smoking) may also impact ocular health (Wolkoff et al., 2003; Rozanova et al., 2009).

#### 2.4.1 Indoor meteorological factors

DED has been associated with indoor temperature, RH, and air pollution (organic and inorganic). In one American study, 396 office workers working on two floors of the same building had ocular pain assessed weekly via a questionnaire (scale 0–25)—a 1°C decrease in temperature was associated with an increased severity of dryness, itching, and irritation [OR = −1.11 (per unit decrease in temperature), 95% CI = −1.76 to −0.47; *p* < 0.005] (Mendell et al., 2002). Next, similar to outdoor studies, low RH indoors has also been implicated in DED. In a Finnish study, 290 office workers located in two wings of the same building were crossed over between high humidity conditions (30%–40% RH) and “natural” conditions (20%–30%) for 3 weeks each (6 weeks total). Daily ocular pain symptoms (Likert 0–3) were worse on average while working in the low-RH conditions (0.39 vs. 0.35; *p* < 0.05) (Reinikainen et al., 1992). Similar findings were noted in a geographically diverse population—a study of 44 individuals in New Zealand had subjects work with and without a desktop humidifier (which increased RH by 5.4% ± 5.0%). This study found that 36% of participants noted improved ocular comfort scores while working with a humidifier, as compared to 5% in the non-humidifier group; *p* < 0.001) (Wang et al., 2017).

Studies focusing on at-home air PM have aligned with findings focusing on outdoor air pollution. Specifically, an American study of 97 individuals found that a 1 unit increase in PM_2.5_ was associated with increased OSDI (β = 0.59, 95% CI = 0.58 to 2.59; *p* = 0.002) and reduced tear production (Schirmer’s: β = −0.67, 95% CI = 0.75 to −0.03; *p* = 0.04) (Huang et al., 2020). In addition to these factors, building-related factors may also relate to DED—an American study evaluated the short-term effects of 88 subjects working in an older building (with a higher concentration of airborne PM (24,436 particles ≥0.5 μm/ft^3^) as compared to 102 subjects working in a newer building (12,313 particles ≥0.5 μm/ft^3^).

Like with outdoor air, few studies have examined indoor air bioaerosols and how they relate to ocular health. One American VA-based study examined the relationship with ocular health in 157 individuals seen at a VA eye clinic between October 2017 and October 2019. This study examined microbial presence in indoor air via bioaerosol concentrations (CFUs). Positive associations were noted between indoor air microbial load and the amount of corneal epithelial disruption (OR = 28.07, 95% CI = 1.8 to 443.8; *p* < 0.05) as well as with meibomian dropout (OR = 39.6, 95% CI = 1.8 to 875.2; *p* < 0.05). As expected, inter-meteorological relationships were noted; a 1% increase in RH was associated with a 3% increase in CFUs (OR = 0.03, 95% CI = 0.01 to 0.04; *p* < 0.001) (Rock et al., 2021).

Short-term exposures have also been studied as they relate to DED. An American study questioned 88 individuals as they left an older building (with a higher concentration of airborne PM (24,436 particles ≥0.5 μm/ft^3^) as compared to 102 subjects who left a newer building (12,313 particles ≥0.5 μm/ft^3^). When adjusting for other variables (e.g., building and time interaction), there was a 1% increase in the odds of reporting worsening DED symptoms per hour spent in the older versus newer building (OR = 1.01; 95% CI = 1.00 to 1.02; *p* < 0.05). In multivariate analyses, subjects working in the older building for longer periods (upwards of 3 h) were more likely to report pain (OR 3.89, 95% CI = 1.21 to 12.5; *p* < 0.05) than those working in the newer building (Idarraga et al., 2020).

Overall, the literature supports findings that are similar to what has been noted with respect to outdoor exposures, as various indoor exposures have been found to relate to various aspects of DED.

#### 2.4.2 Behavioral factors

##### 2.4.2.1 Smoking

Smoking is a behavioral factor that has been connected to DED, among other ocular conditions, including macular degeneration, glaucoma, and cataracts (Makrynioti et al., 2020). Smoke exposure can lead to tear film instability, secondary to a direct irritant action, through free-radical formation or the promotion of lipid peroxidation at the tear film (Sahai and Malik, 2005; Sayin et al., 2014). Studying this question with a focus on e-cigarettes, an American study examined 49 e-cigarette flavoring liquids and analyzed ROS production (via electron paramagnetic resonance (EPR)) as well as synthetic lipid peroxidation *in vitro* (analyzed for secondary lipid oxidation products using a thiobarbituric acid reactive substances (TBARS) assay kit). The study found that 43% of the e-cigarette flavorants analyzed led to a significant increase in free-radical production as compared to a flavor-free liquid (PG:GLY) (specific data not provided; *p* < 0.05 each). In addition, the effects of these liquids on lipid peroxidation were also measured *in vitro*, and significant increases in lipid peroxidation were noted for several flavorants, especially those that contained linalool (4 mg/mL), piperonal (1.6 mg/mL), and citral (4 mg/mL) (257%, 197%, and 205% increase in peroxidation rate vs. PG:GLY, respectively; specific data not provided; *p* < 0.05) (Bitzer et al., 2018). For reference, similar lipid peroxidase abnormalities have been noted in non-smokers with DED (95), suggesting that similar downstream mechanisms of DED can be caused by a variety of insults.

Epidemiological studies have been mixed with respect to the impact of smoking on ocular health. Some studies have found positive relationships between smoking and DED—in a Turkish study of 49 smokers and 53 non-smokers, tear stability (TBUT: 8.24 ± 2.39 s vs.11.15 ± 1.94 s; *p* < 0.0001) and tear production (Schirmer’s 13.30 ± 4.63 vs. 15.45 ± 4.11; *p* = 0.02) were both decreased in the smoking group. However, the values were still within normal ranges in both groups (Sayin et al., 2014). Supporting these findings, the Beaver Dam study of 3,583 individuals found that DED symptoms were present in 534 patients (14.4%) and that both past (OR = 1.22, 95% CI = 0.97 to 1.52; *p* < 0.05) and current smoking status (OR = 1.82, 95% CI = 1.36 to 2.46; *p* < 0.05) acted as risk factors for symptom presence (Moss et al., 2000). Other studies, however, have not found smoking to be a risk factor for DED—a meta-analysis of 10 studies (two cohort and eight cross-sectional studies) reported no relationship between DED diagnosis and a smoking history, when considering the impact of age and gender (OR = 1.16, 95% CI = 0.83 to 1.64; *p* = 0.38). However, the same study presented a subsequent sensitivity analysis in which only general (non-hospital) populations were included, and in this sub-analysis, the association became significant (OR = 1.50, 95% CI = 1.08 to 2.09; *p* = 0.02) (Xu et al., 2016).

In summary, the effects of smoking on DED are not entirely understood. While studies have demonstrated the direct negative effects of smoking on ocular health *in vitro*, results have been mixed when examined on the epidemiological level. Of growing interest are the health effects of other forms of smoking, such as vaping, for which preliminary studies have also demonstrated toxic effects on ocular health (Isa et al., 2019; Martheswaran et al., 2021).

##### 2.4.2.2 Video display units

The impact of office work has been studied with respect to ocular disease (Huang et al., 2020), with the focus centered on the use of video display units (VDUs; e.g., computer screens) and reading tasks suggesting altered blink rates due to these tasks negatively affecting ocular health (Wolkoff, 2020). A Saudi study demonstrated a time-dependent positive association between ocular discomfort scores and visual tasks—in this study, 40 healthy men who read from a book and an electronic tablet for 15 min each found that the blink rate decreased significantly under both reading conditions (19.74 ± 9.12 blinks/min at baseline to 11.35 ± 0.20 and 14.93 ± 10.90 blinks/min for book and a tablet; *p* < 0.05 each). Concurrently, ocular discomfort scores [via a visual analog scale (VAS)] increased significantly from baseline values at all time intervals (5, 10, and 15 min) during both forms of reading. While still being explored, studies suggest that prolonged VDU use has a negative impact on ocular health.

### 2.5 Molecular mechanisms of injury

As previously stated, several exposures have been linked to ocular surface disorders like DED. However, the mechanisms that link a specific environmental insult to a specific facet of DED have not been fully elucidated. Some potential mechanisms include hyperactivation of pro-inflammatory cytokines and reactive oxygen species (ROS) (Zheng et al., 2014; Dogru et al., 2018; Ma et al., 2021), pathological apoptosis of epithelia (corneal and conjunctival) (Yeh et al., 2003; Stern et al., 2004), impaired activation of protective autophagy mechanisms (Wang et al., 2019; Liu et al., 2020), and tear film unit glandular dysfunction [e.g., lacrimal and meibomian dysfunction as a result of immune cell infiltration (Hikichi et al., 1993) and hyperkeratinization] (Jester et al., 1981; Yu et al., 2021).

Only a few studies have examined the molecular mechanisms that underlie the impact of adverse ambient conditions (RH, temperature) on the eye, with most focusing on animal or *in vitro* human cell models. One Canadian study examined tear cytokine levels after an incident of desiccating stress in volunteers with known DED (*n* = 8, diagnostic criteria not provided) and healthy controls (*n* = 8)—individuals sat in an environmental chamber with a controlled temperature (23°C ± 3°C), relative humidity (10% ± 3%), and air velocity (3–5 ft/s) for 180 min. Basal tears were collected before and after exposure to the low-RH environment, and tears were analyzed for cytokines (via V-plex assay). Individuals with DED had higher baseline IL-2 levels than controls (1.11 ± 0.83 pg/mL vs. 0.45 ± 0.37 pg/mL; *p* < 0.05). Post-exposure, IL-2 levels significantly increased in the DED group compared to baseline (1.57 ± 0.91 pg/mL vs. 1.11 ± 0.83 pg/mL; *p* < 0.05). On the other hand, no significant changes were noted in the control group after exposure (0.45 ± 0.37 pg/mL vs. 0.47 ± 0.15 pg/mL, *p* > 0.05) (Subbaraman et al., 2014). In summary, preliminary findings suggest that inflammatory mediators may link desiccating stress to tear abnormalities, with individuals differentially impacted based on baseline disease status.

A larger body of literature has focused on the molecular consequences of air pollution (PM and reactive gases). One study exposed mice to PM_10_ (50 µL PM_10_ eye drop four times daily for 14 days to the right eye). Expression of pro-inflammatory molecules in the cornea (TNF-α; NF-κB) increased when compared to non-exposed eyes (specific data not provided; *p* < 0.05 for each). Furthermore, an increased level of apoptosis was noted in the corneal superficial and basal epithelia in the PM_10_-treated group (specific data not provided; *p* < 0.05 for each) (Li et al., 2017). Other *in vitro* human (Tau et al., 2013) and animal model studies (Li et al., 2019) have similarly reported increased tear cytokines after exposure to air pollutants. Another noted mechanism is corneal epithelial oxidative stress— an *in vitro* Chinese study that studied the effects of air pollution (up to 320 μg/100 μL of PM) on human corneal epithelial cells found a dose–response relationship between PM concentration and oxidative stress (via 8-hydroxy-2' -deoxyguanosine (8OHdG): 214 ± 6.50 pg/mL with 5 μg/100 μL of PM vs. 400 ± 38.8 pg/mL with 80 μg/100 μL of PM; *p* < 0.005) (Xiang et al., 2016). Finally, altered cell autophagy has also been noted due to PM—an *in vitro* study found that human corneal epithelial cells exposed to PM_2.5_ (50 μg/mL) had changed to autophagy; increased autophagosome formation was noted via immunofluorescence of epithelial cell LC3B (an autophagy-associated marker; ∼80% of total cells expressing autophagy post exposure vs. ∼45% in non-exposed; *p* < 0.01). Interestingly, this effect did not occur linearly. Western blot analysis showed that the expression of LC3B decreased during the first 4 h of exposure and then slowly returned to the baseline before increasing with longer exposure periods (Fu et al., 2017). This suggests a time-dependent role in autophagy that requires further study.

Other studies have focused on molecular mechanisms related to reactive gas exposure, like O_3_. One animal study exposed mice to O_3_ (0.5 or 2.0 ppm of O_3_ for 3 h in a whole-body exposure chamber) and noted conjunctival goblet cell damage on IVCM and a dose-dependent increase in tear cytokines (via BD cytometric bead array) post exposure as compared to baseline. Specifically, IL-1β, IL-6, IL-17, interferon (IFN)-γ, and NF-κB translocation and transcriptional activity levels all significantly increased at 1 week and 4 weeks after exposure in both experimental groups (specific data not provided; *p* < 0.05 for each) (Lee et al., 2013). In summary, several molecular pathways of injury have been attributed to toxic exposure to air pollution and reactive gases, including proinflammatory cytokine release, corneal oxidative stress, and alteration in normal apoptosis and autophagy mechanisms.

Molecular mechanisms have also been studied for smoking. One rat model examined corneal health after cigarette smoke exposure via a smoking chamber (six daily episodes, each 3 h long to 300 mL of x 5 days). Immunohistochemical analysis reported increased oxidative stress in the corneal epithelium and lacrimal glands after exposure (via 8OhdG: specific data not provided; *p* < 0.05 for each) (Higuchi et al., 2011). This has also been investigated in humans—a Japanese study exposed 12 healthy individuals to smoking in a controlled chamber for 5 min. Increased tear inflammatory cytokines, most notably IL-6, were reported at both 5 min and 24 h post exposure compared to pre-exposure (specific data not provided; *p* < 0.05 for each). In addition, tear abnormalities were noted in the form of increased tear evaporation (post: 3.34 ± 2.04 (10^–7^) g/cm^2^/s vs. pre: 1.84 ± 1.19 (10^–7^) g/cm^2^/s; *p* < 0.05) (Rummenie et al., 2008). The authors hypothesized that the change in evaporation was related to lipid layer peroxidation and damage, as this has been observed after cigarette smoking in other human studies (Choi et al., 2016; Bitzer et al., 2018).

Shared molecular pathways have also been found that link ocular allergy and DED (Proud et al., 1990; Albrecht and Dittrich, 2018). Mucin layer dysfunction has been implicated in both ocular allergy and DED independently (Davidson and Kuonen, 2004; Rabensteiner et al., 2019). The mucus layer, adjacent to the corneal epithelium, functions as part of the tear film to lubricate and protect the cornea, anchor the aqueous layer to the corneal epithelium, and modulate shearing forces, and dysfunction in this layer has been demonstrated in patients with known DED. In particular, studies have reported reduced or altered expression of mucins in the bulbar and tarsal conjunctiva of individuals with DED (Pflugfelder et al., 1997; Danjo et al., 1998). Demonstrating this finding in ocular allergy, a Japanese study that examined 18 individuals with atopic keratoconjunctivitis and 14 controls found alterations in corneal epithelium mucin transcription in atopic eyes. Specifically, increased MUC16 expression (501 copies/ng vs. 143 copies/ng in controls; *p* = 0.001) and decreased MUC5AC expression (311 copies/ng vs. 1,006 copies/ng in controls; *p* = 0.001) were reported (Dogru et al., 2008). Overall, this suggests pathologic changes to mucus layer protein expression in a similar manner to those observed in individuals with DED.

In summary, the research has implicated several molecular mechanisms, including inflammation, oxidative stress, and altered apoptosis and autophagy, as underlying causes that may explain the association between toxic environmental and behavioral exposures and risk for ocular diseases like DED. Given the lack of studies examining these relationships, especially with respect to variables such as temperature and allergen exposure, further research is needed to fully understand these relationships.

### 2.6 Mitigation strategies

Several mitigation strategies exist that can target outdoor and indoor environmental conditions, with varying levels of difficulty and cost. For patients with severe or refractory disease, environmental modulation should be considered. Understandably, mitigation strategies for the outdoor environment are difficult. With regards to direct contact exposures (e.g., pollutants and aeroallergens), providers have recommended frequent hand washing, wearing wrap-around glasses or goggles, the use of pollen screens, and tracking local forecasted levels to mitigate outdoor exposures (Bergmann et al., 2021).

Mitigation strategies for the indoor environment are more plausible, given that the space is smaller and thus more controllable. Options include maintaining temperature and humidity in the “Goldilocks” zone (with the use of air conditioning and humidifiers). According to EPA guidelines, the optimal indoor RH should be set between 30% and 50%. According to the American Society of Heating, Refrigerating, and Air-Conditioning Engineers, the indoor temperature should be set between 20°C and 25°C (Abdul-Wahab et al., 2015). Managing indoor sources of pollution is another important strategy. Steps to reduce indoor PM levels include replacing filters on central heating and cooling systems, installing air purifiers, and avoiding unvented stoves and fireplaces (Mandell et al., 2020). In addition to this, removing sources of mold growth (paper, sheetrock (drywall), and carpet) is also a possible strategy (Vance et al., 2016). While not studied directly in DED, similar environmental controls have been found to be beneficial in 937 children with atopic asthma. In a US-based trial, caretakers in the intervention group were asked to perform mitigation behaviors that were tailored to each child’s skin-test-sensitization results for 1 year. These included high-efficiency air purifiers, allergen-impermeable covers on mattresses and pillows, and specific allergy interventions such as pest control for children with cockroach allergies. In the control group, no interventions were undertaken. Families were contacted every 2 months and asked about the number of days with symptoms such as wheezing, chest tightness, cough, disturbed sleep, or decreased playtime due to asthma in the last 2 weeks before the phone interview. The group that underwent interventions had fewer active symptom days than controls (3.39 ± 0.12 days vs. 4.20 ± 0.12 in a 14-day period; *p* < 0.001) (Morgan et al., 2004). Similar approaches can be considered for DED.

## 3 Conclusion

**FIGURE 1:**
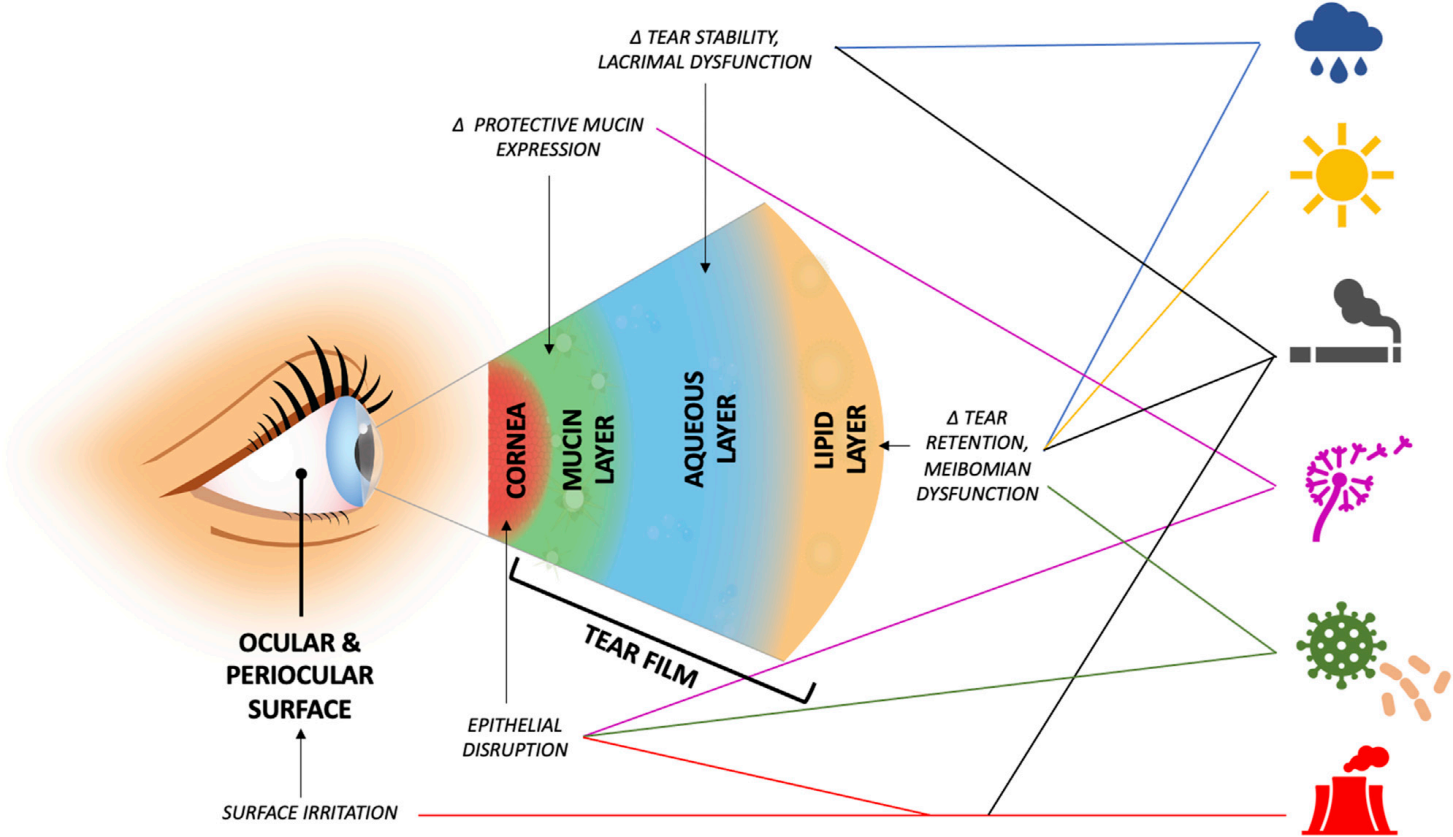
Suggested areas of dysfunction and possible underlying mechanisms that predispose to ocular surface disease by environmental exposure (exposures, top to bottom, by icon: relative humidity, ambient temperature, smoking, aeroallergens, bioaerosols, air pollutants, and reactive gases). Δ = alteration in.

Our Review highlights that environmental and behavioral exposures can impact the risk of DED diagnosis, symptoms, and signs, both in individuals with pre-existing DED and in healthy individuals. The studies summarized in this article suggest positive relationships between DED and weather extremes and air pollution, including PM, gases, allergens, and bioaerosols. In addition, links to behavioral factors like smoking have been reported, albeit with inconsistency in findings across studies. Data suggest that these environmental components may contribute to aspects of DED through a variety of molecular mechanisms (Table 1). The pathophysiologic mechanisms that underlie the noted associations require further study to elucidate causal pathways, but several theories have been included in the Review (Figure 1). Given these findings, we suggest mitigation factors should be considered in appropriate patients (Alves et al., 2014); indoor factors such as air filters to minimize pollutant and allergen levels or tighter control of indoor RH and temperature may be the most cost-effective solutions for those most at risk. In the meantime, these associations can be incorporated into clinical practice by discussing exposure avoidance and/or mitigation for susceptible patients (Rozanova et al., 2009).

**TABLE 1:** Summary of hypothesized mechanisms underlying toxic environmental exposures that may predispose to DED.

Environmental exposure	Hypothesized mechanism
Temperature	Altered tear film lipid layer and tear film thickness (Nagymihályi et al., 2004)
Relative humidity (RH)	Altered tear film lipid layer and tear evaporation and increased tear inflammatory cytokines (Abusharha and Pearce, 2013; Peng et al., 2014; Subbaraman et al., 2014)
Air pollution and particulate matter (PM)	Direct surface irritant (larger molecules), corneal oxidative stress (smaller molecules), increased tear inflammatory cytokines, increased corneal epithelial apoptosis, and altered cell autophagy (Xiang et al., 2016; Fu et al., 2017; Li et al., 2017; Mandell et al., 2020)
Reactive gases	Corneal oxidative stress and increased tear inflammatory cytokines (Lee et al., 2013; Mandell et al., 2020)
Aeroallergens	Corneal epithelial disruption, corneal oxidative stress (IgE-mediated), and altered glandular mucins (Dogru et al., 2008; Rabensteiner et al., 2019)
Smoke exposure	Direct surface irritant, altered tear film lipid layer, excessive and reflexive tearing, corneal oxidative stress, and increased tear inflammatory cytokines (Sahai and Malik., 2005; Rummenie et al., 2008; Higuchi et al., 2011; Sayin et al., 2014)
Bioaerosols	Corneal epithelial disruption and promotion of Meibomian gland disease (Rock et al.,2021)
